# The impact of not having a primary care provider on emergency department utilization and hospitalizations before and during COVID-19: A novel retrospective cohort study linking primary care waitlists with administrative billing data.

**DOI:** 10.23889/ijpds.v7i3.1932

**Published:** 2022-08-25

**Authors:** Emily Gard Marshall, David Stock, Richard Buote

**Affiliations:** 1 Primary Care Research Unit, Department of Family Medicine, Dalhousie University

## Objectives

Primary care attachment improves access, chronic disease prevention and management. Growing proportions of Canadians are unattached and registering with provincial primary care waitlists for family doctors or nurse practitioners. We compare emergency department utilization and hospitalization by waitlist registration status both before and during the first two waves of COVID-19.

## Approach

This study is the first to link a provincial primary care waitlist with routinely collected administrative billing data. Access and linking required collaboration and processes to establish permissions and rigour. A descriptive cohort design estimates quarterly population-based rates of emergency department utilization and ambulatory care sensitive condition (ACSC) hospitalizations among persons on and off the waitlist between Jan/01/2017-Dec/24/2020. Emergency department utilization and ACSC hospitalization rates by current waitlist status were quantified from physician claims and hospitalization data. Relative differences during COVID-19 first (Q2 2020; April-June 2020) and second waves (Q4 2020; October-December 2020) were compared with the previous year.

## Results

Centralized waitlist and administrative billing data were successfully linked. During the study period, 100,867 primary care-eligible Nova Scotians (10.1% of the population) were on the waitlist. Those on the waitlist had higher emergency department utilization each quarter, and more ACSC hospitalizations for most quarters, than those not on the waitlist. Emergency department utilization was higher for individuals ≥65 years and females; lowest during the first two COVID-19 waves; and differed more by waitlist status for those <65 years. Emergency department contacts and ACSC hospitalizations decreased during COVID-19 relative to the previous year. Emergency department utilization during COVID-19 was lower compared to analogous previous year quarters and this relative difference was more pronounced for those on the waitlist during the second wave of COVID-19.

## Conclusion

Linking novel data sources identified that Nova Scotians seeking primary care attachment utilize hospital-based services more frequently than those not on the waitlist. Both groups had lower utilization during the COVID-19 pandemic than the year before. The degree to which forgone services produces downstream health burden remains to be seen.

